# Rate-Distortion Analysis of Distributed Indirect Source Coding

**DOI:** 10.3390/e27080844

**Published:** 2025-08-08

**Authors:** Jiancheng Tang, Qianqian Yang

**Affiliations:** College of information Science and Electronic Engineering, Zhejiang University, Hangzhou 310007, China; jianchengtang@zju.edu.cn

**Keywords:** semantic communication, distributed source coding, rate-distortion theory, side information, Blahut–Arimoto algorithm

## Abstract

Motivated by task-oriented semantic communication and distributed learning systems, this paper studies a distributed indirect source coding problem where *M* correlated sources are independently encoded for a central decoder. The decoder has access to correlated side information in addition to the messages received from the encoders and aims to recover a latent random variable under a given distortion constraint rather than recovering the sources themselves. We characterize the exact rate-distortion function for the case where the sources are conditionally independent given the side information. Furthermore, we develop a distributed Blahut–Arimoto (BA) algorithm to numerically compute the rate-distortion function. Numerical examples are provided to demonstrate the effectiveness of the proposed approach in calculating the rate-distortion region.

## 1. Introduction

**Figure 1:**
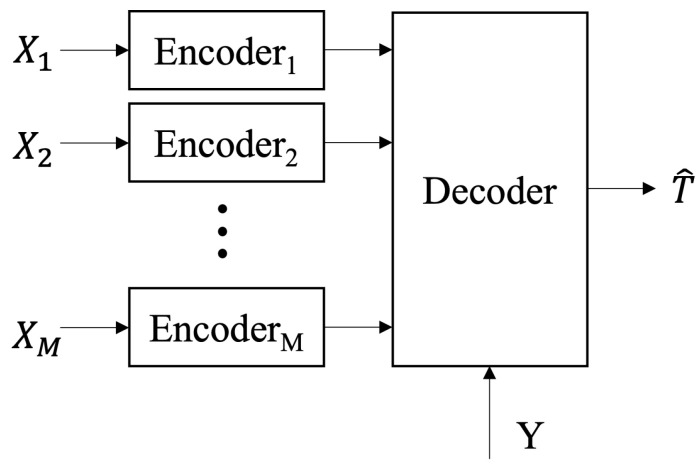
Distributed remote compression of a latent variable with *M* correlated sources at distributed transmitters and side information at the receiver.

Consider the multiterminal source coding setup as shown in Figure 1. Let $\left(T,X_{1},\ldots ,X_{M},Y\right)$$∼p(t,x_{1},\ldots ,x_{M},y)$ be a discrete memoryless source (DMS) taking values in the finite alphabets $T\times X_{1}\times \cdots \times X_{M}\times Y$ according to a fixed and known probability distribution $p(t,x_{1},\ldots ,x_{M},y)$. In this setup, the encoder m,m∈M:={1,…,M} has local observations $X_{m}^{n}:=\left(X_{m}^{1},\ldots ,X_{m}^{n}\right)$. The agents independently encode their observations into binary sequences at rates $\left\{R_{1},\ldots ,R_{M}\right\}$ bits per input symbol, respectively. The decoder with side information $Y^{n}=\left(Y_{1},\ldots ,Y_{n}\right)$ aims to recover some task-oriented latent information $T^{n}:=\left(T_{1},\ldots ,T_{n}\right)$ which is correlated with $\left(X_{1}^{n},\ldots ,X_{M}^{n}\right)$, but it is not observed directly by any of the encoders. We are interested in the lossy reconstruction of $T^{n}$ with the average distortion measured by $E\left[\frac{1}{n}\sum_{i=1}^{n}d\left(T_{i},{\hat{T}}_{i}\right)\right]$ for some prescribed single-letter distortion measure d(·,·). A formal $\left(2^{nR_{1}},\ldots ,2^{nR_{M}},n\right)$ rate-distortion code for this setup consists of the following:

- *M* independent encoders, where encoder m∈M assigns an index $s_{m}\left(x_{m}^{n}\right)\in \left\{1,\ldots ,2^{nR_{m}}\right\}$ to each sequence $x_{m}^{n}\in X_{m}^{n}$;
- A decoder that produces the estimate ${\hat{t}}^{n}\left(s_{1},\ldots ,s_{M},y^{n}\right)\in T^{n}$ to each index tuple $\left(s_{1},\ldots ,s_{M}\right)$ and side information $y^{n}\in Y^{n}$.

A rate tuple $\left(R_{1},\ldots ,R_{M}\right)$ is said to be achievable with the distortion measure d(·,·) and the distortion value *D* if there exists a sequence of $\left(2^{nR_{1}},\ldots ,2^{nR_{M}},n\right)$ codes that satisfy

(1) $$\begin{array}{c} \underset{n\to \infty }{\lim\sup}E\left[\frac{1}{n}\sum_{i=1}^{n}d\left(T_{i},{\hat{T}}_{i}\right)\right]\leq D. \end{array}$$

The rate-distortion region $R_{X_{1},\ldots ,X_{m}|Y}^{*}\left(D\right)$ for this distributed source coding problem is the closure of the set of all achievable rate tuples $\left(R_{1},\ldots ,R_{M}\right)$ that permit the reconstruction of the latent variable $T^{n}$ within the average distortion constraint *D*.

The problem as illustrated in Figure 1 is motivated by semantic/task-oriented communication and distributed learning problems. In semantic/task-oriented communication [1], the decoder only needs to reconstruct some task-oriented information implied by the sources. For instance, it might extract hidden features from a scene captured by multiple cameras positioned at various angles. Here, $T_{i}$ may also be a deterministic function of the source samples $\left(X_{1,i},\ldots ,X_{M,i}\right)$, which then reduces to the problem of lossy distributed function computation [2,3,4]. A similar problem also arises in distributed training. Consider $Y^{n}$ as the global model available at the server at an iteration of a federated learning process and $\left(X_{1}^{n},\ldots ,X_{M}^{n}\right)$ as the independent correlated versions of this model after downlink transmission and local training. The server aims to recover the updated global model, $T^{n}$, based on the messages received from all *M* clients. It is often assumed that the global model is transmitted to the clients intact, but in practical scenarios where downlink communication is limited, the clients may receive noisy or compressed versions of the global model [5,6,7].

For the case of M=1, the problem reduces to the remote compression in a point-to-point scenario with side information available at the decoder. In [8,9], the authors studied this problem without the correlated side information at the receiver, which is motivated in the context of semantic communication. This problem is known in the literature as the remote rate-distortion problem [10,11], and the rate-distortion trade-off is fully characterized in the general case. The authors studied this trade-off in detail for specific source distributions in [8]. Similarly, the authors of [12] characterized the remote rate-distortion trade-off when correlated side information is available both at the encoder and decoder. Our problem for M=1 can be solved by combining the remote rate-distortion problem with the classical Wyner–Ziv rate-distortion function [13,14].

The rate-distortion region for the multi-terminal version of the remote rate-distortion problem considered here remains open. Wagner et al. developed an improved outer bound for a general multiterminal source model [15]. Sung et al. proposed an achievable rate region for the distributed lossy computation problem without giving an conclusive rate-distortion function [16]. Gwanmo et al. considered a special case in which the sources are independent and derived a single-letter expression for the rate-distortion region [17]. Gastpar [18] considered the lossy compression of the source sequences in the presence of side information at the receiver. He characterized the rate-distortion region for the special case, in which $X_{i}$ values are conditionally independent given the side information.

To provide a performance reference for practical coding schemes, it is necessary not only to characterize the exact theoretical expression for the rate-distortion region but also to calculate the rate-distortion region for a given distribution and a specific distortion metric. In the traditional single-source direct scenario, determining the rate-distortion function involves solving a convex optimization problem, which can be addressed using the globally convergent iterative Blahut–Arimoto algorithm, as discussed in [17]. In this paper, we are interested in computing the rate-distortion region $R_{X_{1},\ldots ,X_{m}|Y}^{*}\left(D\right)$ for the general distributed coding problem. We pay particular attention to the special case in which the sources are conditionally independent given the side information, which is motivated by the aforementioned examples. For the sake of brevity of the presentation, we set M=3 in this paper with the understanding that the results can be readily extended to an arbitrary number of sources. To numerically compute the rate-distortion region, we introduce a distributed iterative optimization framework that generalizes the classical Blahut–Arimoto (BA) algorithm. While the standard BA algorithm is designed for single-source point-to-point settings, our framework extends its alternating minimization structure to a distributed scenario with multiple encoders, indirect source reconstruction, and decoder-side side information. This extension enables the computation of rate-distortion regions in settings that are significantly more general than those considered in the existing literature.

In Section 2, we derive an achievable region $R_{a}\left(D\right)\subseteq R_{X_{1},X_{2},X_{3}|Y}^{*}\left(D\right)$. In Section 3, we determine a general outer bound $R_{o}\left(D\right)⊇R_{X_{1},X_{2},X_{3}|Y}^{*}\left(D\right)$. In Section 4, we show that the two regions coincide and the region is optimal when the sources $\left(X_{1},X_{2},X_{3}\right)$ are conditionally independent given the side information *Y*. In Section 5, we develop an alternating minimization framework to calculate the rate-distortion region by generalizing the Blahut–Arimoto (BA) algorithm. In Section 6, we demonstrate the effectiveness of the proposed framework through numerical examples.

## 2. An Achievable Rate Region

In this section, we introduce an achievable rate region $R_{a}\left(D\right)$, which is contained within the goal rate-distortion region $R_{a}\left(D\right)\subseteq R_{X_{1},X_{2},X_{3}|Y}^{*}\left(D\right)$.

**Theorem** **1.** $R_{a}\left(D\right)\subseteq R_{X_{1},X_{2},X_{3}|Y}^{*}\left(D\right)$, where $R_{a}\left(D\right)$ is the set of all rate tuples $\left(R_{1},R_{2},R_{3}\right)$ such that there exists a tuples $\left(W_{1},W_{2},W_{3}\right)$ of discrete random variables with $p\left(w_{1},w_{2},w_{3},x_{1},x_{2},x_{3},y\right)=$$p\left(w_{1}|x_{1}\right)p\left(w_{2}|x_{2}\right)p\left(w_{3}|x_{3}\right)p\left(x_{1},x_{2},x_{3},y\right)$, for which the following conditions are satisfied

(2a) $$\begin{array}{cc} \;\;\;\;\;\;R_{1} & ⩾I\left(X_{1};W_{1}\right)-I\left(W_{1};W_{2},W_{3},Y\right) \end{array}$$

(2b) $$\begin{array}{cc} \;\;\;\;\;\;R_{2} & ⩾I\left(X_{2};W_{2}\right)-I\left(W_{2};W_{1},W_{3},Y\right) \end{array}$$

(2c) $$\begin{array}{cc} \;\;\;\;\;\;R_{3} & ⩾I\left(X_{3};W_{3}\right)-I\left(W_{3};W_{1},W_{2},Y\right) \end{array}$$

(2d) $$\begin{array}{cc} \;\;\;\;\;\;\;\;\;R_{1}+R_{2} & ⩾I\left(X_{1};W_{1}\right)+I\left(X_{2};W_{2}\right)-I\left(W_{1};W_{2},W_{3},Y\right)\\ & -I\left(W_{2};W_{1},W_{3},Y\right)+I\left(W_{1};W_{2}|W_{3},Y\right) \end{array}$$

(2e) $$\begin{array}{cc} \;\;\;\;\;\;\;\;R_{1}+R_{3} & ⩾I\left(X_{1};W_{1}\right)+I\left(X_{3};W_{3}\right)-I\left(W_{1};W_{2},W_{3},Y\right)\\ & -I\left(W_{3};W_{1},W_{2},Y\right)+I\left(W_{1};W_{3}|W_{2},Y\right) \end{array}$$

(2f) $$\begin{array}{cc} \;\;\;\;\;\;\;\;R_{2}+R_{3} & ⩾I\left(X_{2};W_{2}\right)+I\left(X_{3};W_{3}\right)-I\left(W_{2};W_{1},W_{3},Y\right)\\ & -I\left(W_{3};W_{1},W_{2},Y\right)+I\left(W_{2};W_{3}|W_{1},Y\right), \end{array}$$

(2g) $$\begin{array}{cc} \;\;\;R_{1}+R_{2}+R_{3} & ⩾I\left(X_{1};W_{1}\right)+I\left(X_{2};W_{2}\right)+I\left(X_{3};W_{3}\right)\\ & -I\left(W_{1};W_{2},W_{3},Y\right)-I\left(W_{2};W_{1},W_{3},Y\right)\\ & -I\left(W_{3};W_{1},W_{2},Y\right)+I\left(W_{1};W_{2}|W_{3},Y\right)\\ & +I\left(W_{1},W_{2};W_{3}|Y\right), \end{array}$$

*and there exist a decoder $g\left(\cdot \right)$ such that*

(3) $$\begin{array}{c} Ed(T,g(W_{1},W_{2},W_{3},Y))⩽D. \end{array}$$

The auxiliary random variables $W_{1}$, $W_{2}$ and $W_{3}$ serve as intermediate variables in the encoding process, which was introduced to optimize compression efficiency. Ideally, $W_{1}$, $W_{2}$ and $W_{3}$ should carry information about the source $X_{1}$, $X_{2}$ and $X_{3}$, respectively. But this information should be as independent as possible from the side information *Y* in order to avoid redundancy and fully exploit the decoder-side knowledge. This helps minimize the required transmission rate *R*. The rigorous proof of Theorem 1 is provided in Appendix A.

**Corollary** **1.** *The conditions* (25) *of Theorem 1 can be expressed equivalently as*

(4a) $$\begin{array}{cc} \;\;\;\;R_{1} & ⩾I\left(X_{1},X_{2},X_{3};W_{1}|W_{2},W_{3},Y\right) \end{array}$$

(4b) $$\begin{array}{cc} \;\;\;\;R_{2} & ⩾I\left(X_{1},X_{2},X_{3};W_{2}|W_{1},W_{3},Y\right) \end{array}$$

(4c) $$\begin{array}{cc} \;\;\;\;R_{3} & ⩾I\left(X_{1},X_{2},X_{3};W_{3}|W_{1},W_{2},Y\right) \end{array}$$

(4d) $$\begin{array}{cc} \;\;R_{1}+R_{2} & ⩾I\left(X_{1},X_{2},X_{3};W_{1},W_{2}|W_{3},Y\right) \end{array}$$

(4e) $$\begin{array}{cc} \;\;R_{1}+R_{3} & ⩾I\left(X_{1},X_{2},X_{3};W_{1},W_{3}|W_{2},Y\right) \end{array}$$

(4f) $$\begin{array}{cc} \;\;R_{2}+R_{3} & ⩾I\left(X_{1},X_{2},X_{3};W_{2},W_{3}|W_{1},Y\right) \end{array}$$

(4g) $$\begin{array}{cc} R_{1}+R_{2}+R_{3} & ⩾I(X_{1},X_{2},X_{3};W_{1},W_{2},W_{3}|Y) \end{array}$$

**Proof.**  First, we prove $R_{1}⩾I\left(X_{1};W_{1}\right)-I\left(W_{1};W_{2},W_{3},Y\right)=I\left(X_{1},X_{2},X_{3};W_{1}|W_{2},W_{3},Y\right)$. The bound of (4a) can be written as

(5) $$\begin{array}{cc} \; & I\left(X_{1},X_{2},X_{3};W_{1}|W_{2},W_{3},Y\right)\\ \; & =I\left(X_{1};W_{1}|W_{2},W_{3},Y\right)+\underset{=0}{\underset{︸}{I\left(X_{2},X_{3};W_{1}|X_{1},W_{2},W_{3},Y\right)}}, \end{array}$$

where $I\left(X_{2},X_{3};W_{1}|X_{1},W_{2},W_{3},Y\right)=0$ because $\left(X_{2},X_{3},Y\right)$ is conditionally independent of $W_{1}$ for given $X_{1}$. For the first term of the right in (5), we have

(6) $$\begin{array}{cc} \; & I\left(X_{1};W_{1}|W_{2},W_{3},Y\right)+I\left(W_{1};W_{2},W_{3},Y\right)\\ \; & =I\left(W_{1};X_{1},W_{2},W_{3},Y\right)\\ \; & =I\left(W_{1};X_{1}\right)+\underset{=0}{\underset{︸}{I\left(W_{2},W_{3},Y;W_{1}|X_{1}\right)}}, \end{array}$$

where $I\left(W_{2},W_{3},Y;W_{1}|X_{1}\right)=0$ because $\left(W_{2},W_{3},Y\right)$ is conditionally independent of $W_{1}$ given $X_{1}$. Then, we have

(7) $$\begin{array}{cc} \; & I\left(X_{1},X_{2},X_{3};W_{1}|W_{2},W_{3},Y\right)\\ \; & =I\left(X_{1};W_{1}|W_{2},W_{3},Y\right)\\ \; & =I\left(W_{1};X_{1}\right)-I\left(W_{1};W_{2},W_{3},Y\right)\\ \; & \leq R_{1}. \end{array}$$

This completes the proof of (4a). (4b) and (4c) can be proved in the same way.

Then, we prove (4d), the bound of the sum rate $R_{1}+R_{2}$ can be written as

(8a) $$\begin{array}{cc} \; & I\left(X_{1},X_{2},X_{3};W_{1},W_{2}|W_{3},Y\right)\\ \; & =I\left(X_{1};W_{1},W_{2}|W_{3},Y\right)+I\left(X_{2},X_{3};W_{1},W_{2}|W_{3},Y,X_{1}\right) \end{array}$$

(8b) $$\begin{array}{c} \;\;=I\left(X_{1};W_{2}|W_{3},Y\right)+\underset{=I\left(X_{1};W_{1}\right)-I\left(W_{1};W_{2},W_{3},Y\right)\leq R_{1}}{\underset{︸}{I\left(X_{1};W_{1}|W_{2},W_{3},Y\right)}}\\ \;\;\underset{=0}{\underset{︸}{I\left(X_{2},X_{3};W_{1}|W_{3},Y,X_{1}\right)}}+I(X_{2},X_{3};W_{2}|W_{1},W_{3},Y,X_{1}), \end{array}$$

where (8a) and (8b) are obtained by the chain rule of mutual information, $I(X_{2},X_{3};W_{1}|W_{3},$$Y,X_{1})=0$, because $\left(X_{2},X_{3}\right)$ is conditionally independent of $W_{1}$ given $X_{1}$. For the last term in (8b), we have

(9a) $$\begin{array}{c} \;\;\;\;\;\;\;I\left(X_{2},X_{3};W_{2}|W_{1},W_{3},Y,X_{1}\right)+I\left(X_{1};W_{2}|W_{1},W_{3},Y\right) \end{array}$$

(9b) $$\begin{array}{c} =I\left(X_{1},X_{2},X_{3};W_{2}|W_{1},W_{3},Y\right) \end{array}$$

(9c) $$\begin{array}{c} \;\;\;\;\;\;\;\;\;=I\left(X_{2},X_{3};W_{2}|W_{1},W_{3},Y\right)+\underset{=0}{\underset{︸}{I\left(X_{1};W_{2}|W_{1},W_{3},Y,X_{2},X_{3}\right)}} \end{array}$$

(9d) $$\begin{array}{c} \;\;\;\;\;\;\;\;\;=\underset{=I\left(X_{2};W_{2}\right)-I\left(W_{2};W_{1},W_{3},Y\right)\leq R_{2}}{\underset{︸}{I\left(X_{2};W_{2}|W_{1},W_{3},Y\right)}}+\underset{=0}{\underset{︸}{I\left(X_{1},X_{3};W_{2}|W_{1},W_{3},Y,X_{2}\right)}} \end{array}$$

where $I\left(X_{1};W_{2}|W_{1},W_{3},Y,X_{2},X_{3}\right)=0$ because $X_{1}$ is conditionally independent of $W_{2}$ given $X_{2}$, and $I\left(X_{1},X_{3};W_{2}|W_{1},W_{3},Y,X_{2}\right)=0$ because $\left(X_{1},X_{3}\right)$ is conditionally independent of $W_{2}$ given $X_{2}$. Thus, the last term in (8b) can be written as

(10) $$\begin{array}{cc} \; & I\left(X_{2},X_{3};W_{2}|W_{1},W_{3},Y,X_{1}\right)\\ = & \underset{=I\left(X_{2};W_{2}\right)-I\left(W_{2};W_{1},W_{3},Y\right)\leq R_{2}}{\underset{︸}{I\left(X_{2};W_{2}|W_{1},W_{3},Y\right)}}-I\left(X_{1};W_{2}|W_{1},W_{3},Y\right) \end{array}$$

For the last term in the right-hand side, we have

(11) $$\begin{array}{cc} \; & I\left(X_{1};W_{2}|W_{1},W_{3},Y\right)+I\left(W_{1};W_{2}|W_{3},Y\right)\\ \; & =I\left(X_{1},W_{1};W_{2}|W_{3},Y\right)\\ \; & =I\left(X_{1};W_{2}|W_{3},Y\right)+\underset{=0}{\underset{︸}{I\left(W_{1};W_{2}|X_{1},W_{1},W_{3},Y\right)}}, \end{array}$$

thus, the last term in (10) can be written as

(12) $$\begin{array}{cc} \; & I\left(X_{1};W_{2}|W_{1},W_{3},Y\right)\\ = & I\left(X_{1};W_{2}|W_{3},Y\right)-I\left(W_{1};W_{2}|W_{3},Y\right) \end{array}$$

By combining (8), (9), (10) and (12), we have

(13) $$\begin{array}{cc} R_{1}+R_{2} & ⩾I\left(X_{1},X_{2},X_{3};W_{1},W_{2}|W_{3},Y\right)\\ & =I\left(X_{1};W_{1}\right)+I\left(X_{2};W_{2}\right)\\ \; & -I\left(W_{1};W_{2},W_{3},Y\right)-I\left(W_{2};W_{1},W_{3},Y\right)\\ \; & +I\left(W_{1};W_{2}|W_{3},Y\right). \end{array}$$

The rest sum rate bounds in (4) can be proved in the same way, which is omitted here. □

## 3. A General Outer Bound

In this section, we derive a region $R_{o}\left(D\right)$ which contains the goal rate-distortion region $R_{o}\left(D\right)⊇R_{X_{1},X_{2},X_{3}|Y}^{*}\left(D\right)$.

**Theorem** **2.** $R_{o}\left(D\right)⊇R_{X_{1},X_{2},X_{3}|Y}^{*}\left(D\right)$, where $R_{o}\left(D\right)$ is the set of all rate triples $\left(R_{1},R_{2},R_{3}\right)$ such that there exists a triple $\left(W_{1},W_{2},W_{3}\right)$ of discrete random variables with $p\left(w_{1}|x_{1},x_{2},x_{3},y\right)=p\left(w_{1}|x_{1}\right)$, $p\left(w_{2}|x_{1},x_{2},x_{3},y\right)=p\left(w_{2}|x_{2}\right)$ and $p\left(w_{3}|x_{1},x_{2},x_{3},y\right)=p\left(w_{3}|x_{3}\right)$, for which the following conditions are satisfied

(14) $$\begin{array}{c} \begin{array}{cc} R_{1} & ⩾I\left(X_{1},X_{2},X_{3};W_{1}|W_{2},W_{3},Y\right)\\ R_{2} & ⩾I\left(X_{1},X_{2},X_{3};W_{2}|W_{1},W_{3},Y\right)\\ R_{3} & ⩾I\left(X_{1},X_{2},X_{3};W_{3}|W_{1},W_{2},Y\right)\\ R_{1}+R_{2} & ⩾I\left(X_{1},X_{2},X_{3};W_{1},W_{2}|W_{3},Y\right)\\ R_{1}+R_{3} & ⩾I\left(X_{1},X_{2},X_{3};W_{1},W_{3}|W_{2},Y\right)\\ R_{2}+R_{3} & ⩾I\left(X_{1},X_{2},X_{3};W_{2},W_{3}|W_{1},Y\right)\\ R_{1}+R_{2}+R_{3} & ⩾I(X_{1},X_{2},X_{3};W_{1},W_{2},W_{3}|Y) \end{array} \end{array}$$

*and there exists a decoding function $g\left(\cdot \right)$ such that*

(15) $$\begin{array}{c} Ed(T,g_{1}\left(W_{1},W_{2},W_{3},Y\right))⩽D, \end{array}$$

The rigorous proof of Theorem 2 is provided in Appendix B.

While the expressions of the inner bound (4) and the outer bound (14) are the same, these two regions do not coincide because the marginal constrains $p\left(w_{1},w_{2},w_{3},x_{1},x_{2},x_{3},y\right)=$$p\left(w_{1}|x_{1}\right)p\left(w_{2}|x_{2}\right)p\left(w_{3}|x_{3}\right)p\left(x_{1},x_{2},x_{3},y\right)$ in Theorem 1 limit the degree of freedom for choosing the auxiliary random variables $\left(W_{1},W_{2},W_{3}\right)$ compared with the marginal constraints in Theorem 2. In the next section, we will demonstrate that the additional degree of freedom in choosing the auxiliary random variables $\left(W_{1},W_{2},W_{3}\right)$ in Theorem 2 cannot lower the value of the rate-distortion functions.

## 4. Conclusive Rate-Distortion Results

**Corollary** **2.** If $X_{1},X_{2},X_{3}$ are conditionally independent given the side information Y, $R_{a}\left(D\right)\subseteq R_{X_{1},X_{2},|Y}^{*}\left(D\right)$ where $R_{a}\left(D\right)$ is the set of all rate triples $\left(R_{1},R_{2},R_{3}\right)$ such that there exists a triple $\left(W_{1},W_{2},W_{3}\right)$ of random variables with $p\left(w_{1},w_{2},w_{3},x_{1},x_{2},x_{3},y\right)=p\left(w_{1}|x_{1}\right)p\left(w_{2}|x_{2}\right)p\left(w_{3}|x_{3}\right)$$p\left(x_{1}|y\right)p\left(x_{2}|y\right)p\left(x_{3}|y\right)p\left(y\right)$, for which the following conditions are satisfied

(16) $$\begin{array}{cc} R_{1} & ⩾I\left(X_{1};W_{1}\right)-I\left(W_{1};Y\right)\\ R_{2} & ⩾I\left(X_{2};W_{2}\right)-I\left(W_{2};Y\right)\\ R_{3} & ⩾I\left(X_{3};W_{3}\right)-I\left(W_{3};Y\right) \end{array}$$

*and there exist decoding functions $g\left(\cdot \right)$ such that*

(17) $$\begin{array}{c} Ed(T,g(W_{1},W_{2},W_{3},Y))⩽D. \end{array}$$

**Proof.**  Since the joint distribution can be written as

(18) $$\begin{array}{cc} \; & p\left(w_{1},w_{2},w_{3},x_{1},x_{2},x_{3},y\right)\\ & =p\left(w_{1},w_{2},w_{3}|x_{1},x_{2},x_{3},y\right)p\left(x_{1},x_{2},x_{3}|y\right)p\left(y\right)\\ & =p\left(w_{1}|x_{1}\right)p\left(w_{2}|x_{2}\right)p\left(w_{3}|x_{3}\right)p\left(x_{1}|y\right)\\ & \times p\left(x_{2}|y\right)p\left(x_{3}|y\right)p\left(y\right), \end{array}$$

the terms $I\left(W_{1};W_{2}|W_{3},Y\right)$ in the sum rate bound (2d) are 0 because $W_{2}$ is conditionally independent of $W_{1}$. Similarly, the terms $I\left(W_{1};W_{3}|W_{2},Y\right),I\left(W_{2};W_{3}|W_{1},Y\right)$ and $I\left(W_{1};W_{2}|W_{3},Y\right)+$$I\left(W_{1},W_{2};W_{3}|Y\right)$ in the sum rate bound (2e)–(2g) are all 0. Therefore, the sum rate bound can be expressed as the combination of the side bounds and hence can be omitted. Meanwhile, the term $I\left(W_{1};W_{2},W_{3},Y\right)$ in the side bound in (2a) can be written as

(19) $$\begin{array}{c} I\left(W_{1};W_{2},W_{3},Y\right)=I\left(W_{1};Y\right)+\underset{=0}{\underset{︸}{I\left(W_{2},W_{3};W_{1}|Y\right)}}. \end{array}$$

Similarly, we have

(20) $$\begin{array}{c} I\left(W_{2};W_{1},W_{3},Y\right)=I\left(W_{2};Y\right)+\underset{=0}{\underset{︸}{I\left(W_{1},W_{3};W_{2}|Y\right)}}\\ I\left(W_{3};W_{1},W_{2},Y\right)=I\left(W_{3};Y\right)+\underset{=0}{\underset{︸}{I\left(W_{1},W_{2};W_{3}|Y\right)}}. \end{array}$$

This completes the proof Corollary 2. □

**Corollary** **3.** If $X_{1},X_{2},X_{3}$ are conditionally independent given the side information Y, ${R_{o}}^{\prime }\left(D\right)⊇R_{o}\left(D\right)$, and hence ${R_{o}}^{\prime }\left(D\right)⊇R_{X_{1},X_{2},W_{3}|Y}^{*}\left(D\right)$ where ${R_{o}}^{\prime }\left(D\right)$ is the set of all rate triples $\left(R_{1},R_{2},R_{3}\right)$ such that there exists a triple $\left(W_{1},W_{2},W_{3}\right)$ of discrete random variables with $p\left(w_{1}|x_{1},x_{2},w_{3},y\right)=p\left(w_{1}|x_{1}\right)$, $p\left(w_{2}|x_{1},x_{2},w_{3},y\right)=p\left(w_{2}|x_{2}\right)$ and $p\left(w_{3}|x_{1},x_{2},x_{3},y\right)=p\left(w_{3}|x_{3}\right)$, for which the following conditions are satisfied

(21) $$\begin{array}{c} \begin{array}{cc} R_{1} & ⩾I\left(X_{1};W_{1}\right)-I\left(W_{1};Y\right)\\ R_{2} & ⩾I\left(X_{2};W_{2}\right)-I\left(W_{2};Y\right)\\ R_{3} & ⩾I\left(X_{3};W_{3}\right)-I\left(W_{3};Y\right), \end{array} \end{array}$$

*and there exists decoding functions $g\left(\cdot \right)$ such that*

(22) $$\begin{array}{c} Ed(T,g_{1}\left(W_{1},W_{2},W_{3},Y\right))⩽D, \end{array}$$

**Proof.**  First, we can enlarge the region $R_{o}\left(D\right)$ by omitting the sum rate bound in (14). Then, the side rate bounds in (14) can be relaxed as

(23) $$\begin{array}{c} \begin{array}{cc} R_{1} & ⩾I\left(X_{1},X_{2},X_{3};W_{1}|W_{2},W_{3},Y\right)\\ & ⩾I\left(X_{1};W_{1}|W_{2},W_{3},Y\right)+I\left(X_{2},X_{3};W_{1}|W_{2},W_{3},Y,X_{1}\right)\\ & ⩾I\left(X_{1};W_{1}|W_{2},W_{3},Y\right)\\ & =I\left(X_{1};W_{1},W_{2},W_{3}|Y\right)-\underset{=0}{\underset{︸}{I\left(X_{1};W_{2},W_{3}|Y\right)}}. \end{array} \end{array}$$

According to the conditional independence relations, we have $I\left(X_{1};W_{2},W_{3}|Y\right)=0$, and then we have

$$\begin{array}{cc} R_{1} & ⩾I\left(X_{1};W_{1},W_{2},W_{3}|Y\right) \end{array}$$

(24a) $$\begin{array}{c} \;\;\;\;\;\;\;=I\left(X_{1};W_{1}|Y\right)+\underset{=0}{\underset{︸}{I\left(X_{1};W_{2},W_{3}|Y,W_{1}\right)}} \end{array}$$

(24b) $$\begin{array}{c} \;\;\;=I\left(X_{1},Y;W_{1}\right)-I\left(W_{1};Y\right) \end{array}$$

(24c) $$\begin{array}{c} \;\;=I\left(X_{1};W_{1}\right)-I\left(W_{1};Y\right) \end{array}$$

where (24a) is obtained by the condition that $X_{1},X_{2},X_{3}$ are conditionally independent given the side information *Y*, (24b) follows from the chain rule of mutual information and (24c) is derived by the Markov chain $Y-X_{1}-W_{1}$. The same derivation can be applied to $R_{2}$ and $R_{3}$; this proves Corollary 3. □

**Theorem** **3.** *If $X_{1},X_{2},X_{3}$ are conditionally independent given the side information Y,*

(25) $$\begin{array}{c} R_{a}\left(D\right)=R_{o}\left(D\right)=R_{X_{1},X_{2},X_{3}|Y}^{*}\left(D\right). \end{array}$$

**Proof.**  We note that the only difference between $R_{a}\left(D\right)$ and $R_{o}\left(D\right)$ is the degrees of freedom when choosing the auxiliary random variables $\left(W_{1},W_{2},W_{3}\right)$, and all of the mutual information functions in (16) and (21) only depend on the marginal distribution $\left(X_{1},W_{1},Y\right)$, $\left(X_{2},W_{2},Y\right)$ and $\left(X_{3},W_{3},Y\right)$. Randomly choose a certain rate triple $\left(R_{1},R_{2},R_{3}\right)$ with a auxiliary random variable triple $\left(W_{1},W_{2},W_{3}\right)$ meeting the conditions of Corollary 3, and the corresponding joint distribution is given in (18). Then, we construct the auxiliary random variables $\left(W_{1}^{\prime },W_{2}^{\prime },W_{3}^{\prime }\right)$ such that

(26) $$\begin{array}{c} \begin{array}{c} p_{W_{1}^{\prime }|X_{1}}\left(w_{1}|s_{1}\right)=\sum_{w_{2},s_{2},w_{3},s_{3}}p\left(w_{1},w_{2},w_{3}|s_{1},s_{2},s_{3}\right)p\left(s_{2},s_{3}|s_{1}\right)\\ p_{W_{2}^{\prime }|X_{2}}\left(w_{2}|s_{2}\right)=\sum_{w_{1},s_{1},w_{3},s_{3}}p\left(w_{1},w_{2},w_{3}|s_{1},s_{2},s_{3}\right)p\left(s_{1},s_{3}|s_{2}\right)\\ p_{W_{3}^{\prime }|X_{3}}\left(w_{3}|s_{3}\right)=\sum_{w_{1},s_{1},w_{2},s_{2}}p\left(w_{1},w_{2},w_{3}|s_{1},s_{2},s_{3}\right)p\left(s_{1},s_{2}|s_{3}\right). \end{array} \end{array}$$

The joint distribution

(27) $$\begin{array}{c} \begin{array}{cc} \; & p\left(w_{1}^{\prime },w_{2}^{\prime },w_{3}^{\prime },x_{1},x_{2},x_{3},Y\right)\\ & =p\left(w_{1}^{\prime }|x_{1}\right)p\left(w_{2}^{\prime }|x_{2}\right)p\left(w_{3}^{\prime }|x_{3}\right)p\left(x_{1}|y\right)p\left(x_{2}|y\right)p\left(x_{3}|y\right)p\left(y\right) \end{array} \end{array}$$

has the same marginal distributions on $\left(X_{1},W_{1},Y\right)$, $\left(X_{2},W_{2},Y\right)$ and $\left(X_{3},W_{3},Y\right)$. Therefore, the additional degree of freedom for choosing the auxiliary random variables $\left(W_{1},W_{2},W_{3}\right)$ in Corollary 3 cannot lower the value of rate-distortion functions. This proves Theorem 3. The arguments leading to Theorem 3 indicate that the result extends to the *M* sources scenario

(28) $$\begin{array}{c} R_{i}\geq I\left(X_{i};W_{i}\right)-I\left(W_{i};Y\right)\;\mathrm{for}\;\mathrm{all}\;l\in \left\{1,\ldots ,M\right\}. \end{array}$$

□

## 5. Iterative Optimization Framework Based on BA Algorithm

In this section, we present the iterative optimization framework for calculating the rate distortion region. Starting with the standard Lagrange multiplier method, the problem of calculating the rate-distortion region $R_{X_{1},\ldots ,X_{m}|Y}^{*}\left(D\right)$ is equivalent to minimize

(29) $$\sum_{i\in M:=\{1,\ldots ,M\}}I\left(W_{i};X_{i}\right)-I\left(W_{i};Y\right)+\lambda \left(E\left[d\left(T,\hat{T}\right)\right]-D\right)$$

By the definition of mutual information, we can rewrite (29) as

(30) $$\begin{array}{cc} \; & L_{\lambda }\left(Q,q,q^{\prime }\right)\\ & =\sum_{y,\hat{t},x_{i},w_{i},i\in M}p\left(y,x_{i}\right)q_{i}\left(w_{i}|x_{i}\right)q^{\prime }\left(\hat{t}|y,w\right)\log\frac{q_{i}\left(w_{i}|x_{i}\right)}{Q_{i}\left(w_{i}|y\right)}\;\\ & +\lambda \sum_{w,x,t,\hat{t},y}d\left(t,\hat{t}\right)p\left(t,x,y\right)q^{\prime }\left(\hat{t}|y,w\right)\prod_{i\in M}q_{i}\left(w_{i}|x_{i}\right), \end{array}$$

where $Q,q,q^{\prime }$ represent the distributions that need to be iteratively updated, and the vectorized notation Q represents the conditional distribution of the auxiliary variables given *Y*, i.e., $\left[Q_{i}\left(w_{i}|y\right)|w_{i}\in W_{i},y\in Y,i\in M\right]$, q represents the conditional distribution of the auxiliary variables given sources $X_{i}$, $\left[q_{i}\left(w_{i}|x_{i}\right)|w_{i}\in W_{i},x_{i}\in X_{i},i\in M\right]$ and $q^{\prime }$ represents the conditional distribution of the indirect variable *T* given *Y* and auxiliary variables, $q^{\prime }\left(\hat{t}|y,w_{1},\ldots ,w_{M}\right)$.

**Lemma** **1** (Optimization of Q)**.** *For a fixed $Q_{\setminus m},q,q^{\prime }$, the Lagrangian $L_{\lambda }\left(Q,q,q^{\prime }\right)$ is minimized by*

(31) $$Q_{m}^{*}\left(w_{m}|y\right)≜\frac{\sum_{x_{m},\hat{t}}p\left(y,x_{m}\right)q\left(w_{m}|x_{m}\right)q^{\prime }\left(\hat{t}|y,w\right)}{\sum_{x_{m},\hat{t},w_{m}}p\left(y,x_{m}\right)q\left(w_{m}|x_{m}\right)q^{\prime }\left(\hat{t}|y,w\right),}$$

*where $Q_{\setminus m}=\left[Q_{i}\left(w_{i}|y\right)|w_{i}\in W_{i},y\in Y,i\in M\setminus m\right]$.*

**Proof.**  For any $Q_{m}$

(32) $$\begin{array}{c} \begin{array}{cc} \; & L_{\lambda }\left(Q_{m}^{*},q,q^{\prime }\right)-L_{\lambda }\left(Q_{m},q,q^{\prime }\right)\\ & =\sum_{y,\hat{t},x_{m},w_{m}}p\left(y,x_{m}\right)q_{i}\left(w_{m}|x_{m}\right)q^{\prime }\left(\hat{t}|y,w\right)\log\frac{q_{m}\left(w_{m}|x_{m}\right)}{Q_{m}^{*}\left(w_{m}|y\right)}\\ & -\sum_{y,\hat{t},x_{m},w_{m}}p\left(y,x_{m}\right)q_{i}\left(w_{m}|x_{m}\right)q^{\prime }\left(\hat{t}|y,w\right)\log\frac{q_{m}\left(w_{m}|x_{m}\right)}{Q_{m}\left(w_{m}|y\right)}\\ & =\sum_{y,\hat{t},x_{m},w_{m}}p\left(y,x_{m}\right)q_{i}\left(w_{m}|x_{m}\right)q^{\prime }\left(\hat{t}|y,w\right)\log\frac{Q_{m}\left(w_{m}|y\right)}{Q_{m}^{*}\left(w_{m}|y\right)}\\ & \overset{\left(a\right)}{\leq }\sum_{y,\hat{t},x_{m},w_{m}}p\left(y,x_{m}\right)q_{i}\left(w_{m}|x_{m}\right)q^{\prime }\left(\hat{t}|y,w\right)\left(\frac{Q_{m}\left(w_{m}|y\right)}{Q_{m}^{*}\left(w_{m}|y\right)}-1\right)\\ & =0, \end{array} \end{array}$$

where (a) follows from the fact that log(1+x)≤x, and the equality is achieved if $Q_{m}^{*}=Q_{m}$. This completes the proof of Lemma 1. □

**Lemma** **2** (Optimization of q)**.** *For a fixed $Q,q_{\setminus m},q^{\prime }$, the Lagrangian $L_{\lambda }\left(Q,q,q^{\prime }\right)$ is minimized by*

(33) $$q^{*}\left(w_{m}|x_{m}\right)=\frac{\exp\left[\sum_{y}p\left(y|x_{m}\right)\log Q\left(w_{m}|y\right)-\lambda \sum_{w_{\setminus m},x_{\setminus m},t,\hat{t},y}d\left(t,\hat{t}\right)p\left(t,x,y\right)q^{\prime }\left(\hat{t}|y,w\right)q_{\setminus m}\left(w_{\setminus m}|x_{\setminus m}\right)\right]}{\sum_{w_{m}}\exp\left[\sum_{y}p\left(y|x_{m}\right)\log Q\left(w_{m}|y\right)-\lambda \sum_{w_{\setminus m},x_{\setminus m},t,\hat{t},y}d\left(t,\hat{t}\right)p\left(t,x,y\right)q^{\prime }\left(\hat{t}|y,w\right)q_{\setminus m}\left(w_{\setminus m}|x_{\setminus m}\right)\right],}$$

*and the minimum is given by*

(34) $$\begin{array}{cc} \; & L_{\lambda }\left(Q,q_{\setminus m}^{*},q^{\prime }\right)\\ & =\sum_{x_{i},i\in M}p\left(x_{i}\right)\min_{w_{i}}[\sum_{y,\hat{t}}p\left(y|x_{i}\right)q^{\prime }\left(\hat{t}|y,w\right)\log\frac{q^{*}\left(w_{i}|x_{i}\right)}{Q(w_{i}|y)}\\ & +\lambda \sum_{w,x,t,\hat{t},y}d\left(t,\hat{t}\right)p\left(t,x,y\right)q^{\prime }\left(\hat{t}|y,w\right)\prod_{i\in M}q_{i}\left(w_{i}|x_{i}\right)]. \end{array}$$

**Proof.**  For a fixed $Q,q_{\setminus m},q^{\prime }$, the Lagrangian $L_{\lambda }\left(Q,q,q^{\prime }\right)$ is minimized by $q^{*}\left(w_{m}|x_{m}\right)$ if and only if the following Kuhn–Tucker (KT) conditions are satisfied

(35) $${\left.\frac{\partial L_{\lambda }}{\partial q_{m}}\right|}_{q_{m}^{*}}=\gamma ,\;\mathrm{if}\;q^{*}\left(w_{m}|x_{m}\right)>0,$$

and

(36) $${\left.\frac{\partial L_{\lambda }}{\partial q_{m}}\right|}_{q_{m}^{*}}\leq \gamma ,\;\mathrm{if}\;q_{m}^{*}\left(w_{m}|x_{m}\right)=0.$$

Since

(37) $$\begin{array}{cc} \; & \frac{\partial L_{\lambda }}{\partial q_{m}}\\ & =\sum_{x_{m},y}p\left(x_{m},y\right)\left(\log\frac{p(w_{m}|x_{m})}{p(w_{m}|y)}+1\right)\\ & +\lambda \sum_{w,x,t,\hat{t},y}d\left(t,\hat{t}\right)p\left(t,x,y\right)q^{\prime }\left(\hat{t}|y,w\right)q_{\setminus m}\left(u_{\setminus m}|x_{\setminus m}\right), \end{array}$$

the first KT condition (35) becomes

(38) $$\begin{array}{cc} \; & \tilde{\gamma }\\ & =\sum_{x_{m},y}p\left(x_{m},y\right)\left(\log p(w_{m}|x_{m})-\log p(w_{m}|y)\right)\\ & +\lambda \sum_{w_{\setminus m},x_{\setminus m},t,\hat{t},y}d\left(t,\hat{t}\right)p\left(t,x,y\right)q^{\prime }\left(\hat{t}|y,w\right)q_{\setminus m}\left(w_{\setminus m}|x_{\setminus m}\right), \end{array}$$

where

(39) $$q_{\setminus m}\left(w_{\setminus m}|x_{\setminus m}\right)=\prod_{i\in M\setminus m}q_{i}\left(w_{i}|x_{i}\right)$$

and we have

(40) $$\begin{array}{cc} \; & q(w_{m}|x_{m})\\ & =\exp\left(\frac{\tilde{\gamma }}{p(x_{m})}\right)\exp(\sum_{y}p\left(y|x_{m}\right)\log Q\left(w_{m}|y\right)\\ & -\lambda \sum_{w_{\setminus m},x_{\setminus m},t,\hat{t},y}d\left(t,\hat{t}\right)p\left(t,x,y\right)q^{\prime }\left(\hat{t}|y,w\right)q_{\setminus m}\left(w_{\setminus m}|x_{\setminus m}\right)). \end{array}$$

Then, (33) can be obtained after normalizing $q(w_{m}|x_{m})$. □

**Lemma** **3** (Optimization of $q^{\prime }$)**.** *For a fixed Q,q, the Lagrangian $L_{\lambda }\left(Q,q,q^{\prime }\right)$ is minimized by the maximum Bayes detector.*

(41) $$q^{\prime *}\left(\hat{t}|w,y\right)=\left\{\begin{array}{cc} \alpha (w), & \hat{t}\in \arg\min_{\hat{t}\in \hat{T}}E\left[d\left(t,\hat{t}\right)|W=w\right],\\ 0, & \mathrm{otherwise} \end{array}\right.$$

*where α(w) is selected to guarantee*

(42) $$\sum_{\hat{t}}q^{\prime *}\left(\hat{t}|w,y\right)=1$$

*and $E[d\left(t,\hat{t}\right)|W=w]$ denotes*

(43) $$\sum_{w_{\setminus m},x_{\setminus m},t,\hat{t},y}d\left(t,\hat{t}\right)p\left(t,x,y\right)\prod_{i\in M}q_{i}\left(w_{i}|x_{i}\right)$$

**Proof.**  We note that the distortion term in the Lagrangian $L_{\lambda }$ that depends on $q^{\prime }\left(\hat{t}|y,w\right)$ is the mean distortion, which can be minimized by a Bayes detector.

Based on Lemmas 1–3, we have the iterative algorithm for computing the rate-distortion region for the distributed indirect source with decoder side information. For a given i∈M, we iteratively calculate the following (44) and (45) to alternately update $Q_{i}$ and $q_{i}$

(44) $$Q_{i}^{\ell ,k_{i}}=\frac{\sum_{x_{i},\hat{t}}p\left(y,x_{i}\right)q^{\left(\ell ,k_{i}\right)}\left(w_{i}|x_{i}\right)q^{\prime }\left(\hat{t}|y,w\right)}{\sum_{x_{i},\hat{t},w_{i}}p\left(y,x_{i}\right)q^{\left(\ell ,k_{i}\right)}\left(w_{i}|x_{i}\right)q^{\prime }\left(\hat{t}|y,w\right)}$$

(45) $$q_{i}^{\left(l,k_{i}+1\right)}=\frac{\exp\left[\sum_{y}p\left(y|x_{i}\right)\log Q^{\left(l,k_{i}\right)}\left(w_{i}|y\right)-\lambda \sum_{w_{\setminus i},x_{\setminus i},t,\hat{t},y}d\left(t,\hat{t}\right)p\left(t,x,y\right)q^{\prime (\ell ,i)}\left(\hat{t}|y,w\right)q_{\setminus i}^{\left(\ell ,i\right)}\left(w_{\setminus i}|x_{\setminus i}\right)\right]}{\sum_{w_{i}}\exp\left[\sum_{y}p\left(y|x_{i}\right)\log Q^{\left(\ell ,k_{i}\right)}\left(w_{i}|y\right)-\lambda \sum_{w_{\setminus i},x_{\setminus i},t,\hat{t},y}d\left(t,\hat{t}\right)p\left(t,x,y\right)q^{\prime (\ell ,i)}\left(\hat{t}|y,w\right)q_{\setminus i}^{\left(\ell ,i\right)}\left(w_{\setminus i}|x_{\setminus i}\right)\right]}$$

until the Lagrangian $L_{\lambda }$ converges, and the associated $q_{i}$ is

(46) $$q_{i}^{\left(\ell ,*\right)}\left(w_{i}|x_{i}\right)=\lim_{k_{i}\to \infty }q_{i}^{\left(\ell ,k_{i}\right)}\left(w_{i}|x_{i}\right)=:q_{i}^{\left(\ell +1,1\right)}.$$

Then, we update $q^{\prime }\left(\hat{t}|w,y\right)$ according to

(47) $$q^{\prime \ell^{\prime },i^{\prime }}=\left\{\begin{array}{cc} \alpha^{\ell^{\prime },i^{\prime }}\left(w\right), & \hat{t}\in \arg\min_{\hat{t}\in \hat{T}}J^{\left(\ell ,i\right)}\left(\hat{t},w\right),\\ 0, & \mathrm{otherwise} \end{array}\right.$$

with $i^{\prime }=i+1$ and $\ell^{\prime }=\ell$ if i<M and $i^{\prime }=1$ and $\ell^{\prime }=\ell +1$ if i=M, and where $\alpha^{\ell^{\prime },i^{\prime }}\left(w\right)$ is selected to guarantee

(48) $$\sum_{\hat{t}}q^{\prime (\ell^{\prime },i^{\prime })}\left(\hat{t}|w,y\right)=1$$

and

(49) $$\begin{array}{cc} J^{\left(\ell ,i\right)}\left(\hat{t},w\right) & =\sum_{x,t,y}d\left(t,\hat{t}\right)p\left(t,x,y\right)\\ & \times q_{\setminus i}^{\left(\ell ,i\right)}\left(w_{\setminus i}|x_{\setminus i}\right)q_{i}^{\left(\ell ,*\right)}\left(w_{i}|x_{i}\right). \end{array}$$

□

Next, we repeat the process for the next user, i.e., (i←i+1ifi<Mandi←1,ℓ←ℓ+1ifi=M).

Convergence analysis:

The algorithm employs an alternating minimization approach that produces Lagrangian values that are monotonically non-increasing and bounded below, thereby generating a convergent sequence of Lagrangians. Since the rate component of the Lagrangian is convex, and the sum of convex functions remains convex, the Lagrangian will also be convex if the expected distortion is a convex function. As a result, the proposed iterative optimization framework is capable of achieving the global minimum [19].

(50) $$\sum_{w,x,t,\hat{t},y}d\left(t,\hat{t}\right)p\left(t,x,y\right)q^{\prime }\left(\hat{t}|y,w\right)\prod_{i\in M}q_{i}\left(w_{i}|x_{i}\right)$$

We note that the expected distortion (50) includes a product of variables in the optimization process, it is not a linear function, and the Lagrangian may exhibit non-convex behavior. Even when the problem is non-convex, the authors in [20] demonstrate that the BA-based iterative algorithm initialized randomly and followed by selecting the minimum Lagrangian among all converged values can still provide highly effective information-theoretic inner bounds for the rate-distortion region, serving as a benchmark for practical quantization schemes.

## 6. Numerical Examples

In this section, we provide an example to illustrate the proposed iterative algorithms for computing the rate-distortion region of a distributed source coding problem with decoder-side information. As in the problem considered in this paper, distributed edge devices compress their observations $\left\{X_{1},\ldots ,X_{M}\right\}$ and transmit them to a central server (CEO). The central server then aims to recover the indirect information *T* from the received data, utilizing side information *Y*. For the convenience of demonstration, we consider a simple case where M=2 and the sources are binary, i.e., $X_{1}=X_{2}=Y=\left\{0,1\right\}$. The joint distributions, denoted by $Q(x_{1},y)$ and $Q(x_{2},y)$, are given by

(51) $$\begin{array}{c} Q\left(x_{1},y\right)=\frac{\left(1-p_{1}\right)}{2}\delta_{x_{1},y}+\frac{p_{1}}{2}\left(1-\delta_{x_{1},y}\right),\\ Q\left(x_{2},y\right)=\frac{\left(1-p_{2}\right)}{2}\delta_{x_{2},y}+\frac{p_{2}}{2}\left(1-\delta_{x_{2},y}\right), \end{array}$$

where the Kronecker delta function $\delta_{x,y}$ equals 1 when *x* = *y* and 0 otherwise. We can consider *Y* as the input to two different binary symmetric channels (BSCs) with crossover probabilities $p_{1},p_{2}$, respectively, where $0\leq p_{i}\leq \frac{1}{2},i\in \left\{1,2\right\}$. The corresponding outputs of these channels are $X_{1}$ and $X_{2}$. In this example, we set $p_{1}=p_{2}=0.3$.

We also assume that the information of interest is directly the combination of the two distributed sources, i.e., $T=\{X_{1},X_{2}\}$. The distortion measure is given by $d\left(t,\hat{t}\right)=d\left(x_{1},{\hat{x}}_{1}\right)+d\left(x_{2},{\hat{x}}_{2}\right)$, where

(52) $$d\left(x_{i},{\hat{x}}_{i}\right)=\left\{\begin{array}{cc} 0, & x_{i}={\hat{x}}_{i},\\ 1, & x_{i}\neq {\hat{x}}_{i}. \end{array}\right.$$

By applying the proposed iterative optimization framework, we can obtain the optimal transition probability distribution $Q_{i}^{*}\left(w_{i}|y\right),q_{i}^{*}\left(w_{i}|x_{i}\right)$ and $q^{\prime *}\left(\hat{t}|w,y\right)$ that meets a given distortion constraint *D* on $d(t,\hat{t})$, and the corresponding minimum rate $R_{i}$ can be calculated by

(53) $$\begin{array}{c} R_{i}=\sum_{y,x_{i},w_{i}}p\left(y,x_{i}\right)q_{i}^{*}\left(w_{i}|x_{i}\right)\log\frac{q_{i}^{*}\left(w_{i}|x_{i}\right)}{Q_{i}^{*}\left(w_{i}|y\right)}\; \end{array}$$

**Figure 2:**
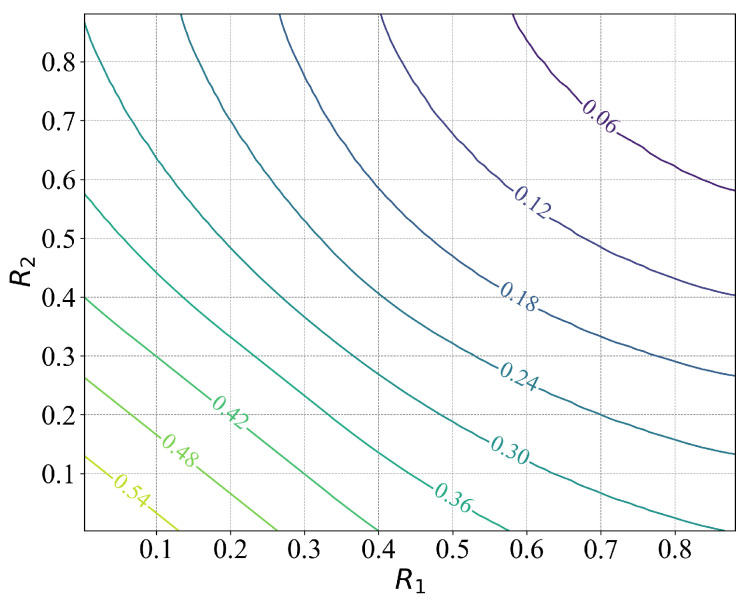
Contour plot of the rate distortion region with two distributed binary sources $\left\{X_{1},X_{2}\right\}$, where the labels on the contours represent the distortion values *D* on $d(t,\hat{t})$.

**Figure 3:**
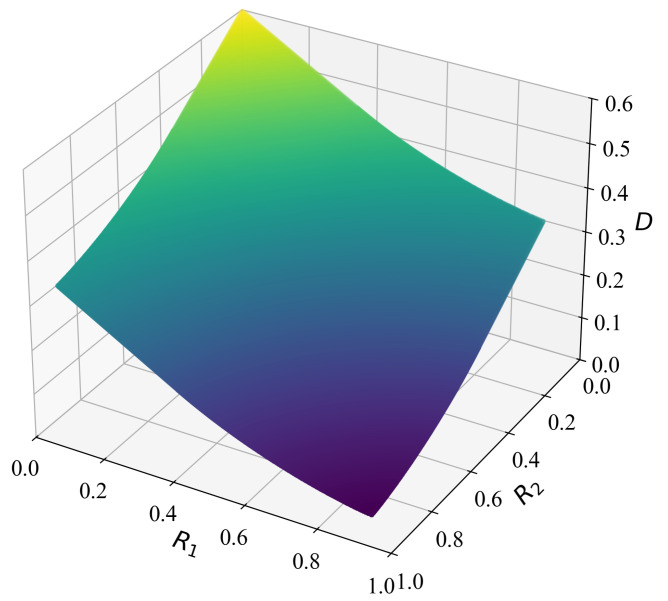
Surface plot of the rate-distortion region.

**Figure 4:**
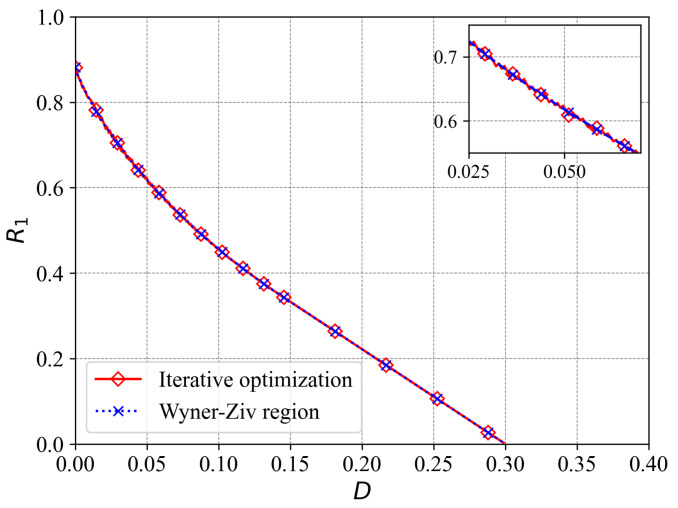
The rate-distortion function for the case when M=1, i.e., the Wyner–Ziv problem.

The contour plot of the rate-distortion region for this scenario is presented in Figure 2, while Figure 3 displays a surface plot depicting the rate-distortion region. We note that when M=1, the considered problem reduces to the traditional point-to-point Wyner–Ziv problem. In Figure 4, we compare the rate-distortion results computed using the proposed approach with the theoretical analysis by Wyner et al. [13]. We observe that the two rate-distortion function curves coincide, demonstrating the effectiveness of the proposed iterative approach for calculating rate distortion.

## 7. Conclusions

This paper explored a variant of the rate-distortion problem motivated by semantic communication and distributed learning systems, where correlated sources are independently encoded for a central decoder to reconstruct the indirect source of interest. In addition to receiving messages from the encoders, the decoder has access to correlated side information and aims to reconstruct the indirect source under a specified distortion constraint. We derived the exact rate-distortion function for the case where the sources are conditionally independent given the side information. Furthermore, we introduced a distributed iterative optimization framework based on the Blahut–Arimoto (BA) algorithm to numerically compute the rate-distortion function. A numerical example has been provided to demonstrate the effectiveness of the proposed approach.

## Data Availability

The original contributions presented in this study are included in the article. Further inquiries can be directed to the corresponding author.

## Appendix A

Here, we provide the rigorous proof of Theorem 1.

**Lemma** **A1** (Extended Markov Lemma)**.** *Let*

(A1) $$\begin{array}{cc} \; & p\left(w_{1},w_{2},w_{3},x_{1},x_{2},x_{3},y\right)\\ & =p\left(w_{1}|x_{1}\right)p\left(w_{2}|x_{2}\right)p\left(w_{3}|x_{3}\right)p\left(x_{1},x_{2},x_{3},y\right). \end{array}$$

*For a fixed $\left(x_{1}^{n},x_{2}^{n},x_{3}^{n}y^{n}\right)\in A_{\epsilon }^{*\left(n\right)}$, $w_{1}^{n}$ , $w_{2}^{n}$ and $w_{3}^{n}$ are drawn from $p(w_{1}|x_{1})$ , $p(w_{2}|x_{2})$ and $p(w_{3}|x_{3})$, respectively. Then*

(A2) $$\begin{array}{c} \lim_{n\to \infty }Pr\left\{\left(w_{1}^{n},w_{2}^{n},w_{3}^{n},x_{1}^{n},x_{2}^{n},x_{3}^{n},y^{n}\right)\in A_{\epsilon }^{*\left(n\right)}\right\}=1. \end{array}$$

**Proof.** According to (A1), we have several Markov chains $W_{1}-X_{1}-\left(X_{2},Y\right)$, $W_{2}-X_{2}-\left(X_{1},Y\right)$, $W_{1}-\left(X_{1},X_{2},Y\right)-W_{2}$. We use the Markov lemma three times: first to derive that $\left(\left(x_{2}^{n},y^{n}\right),x_{1}^{n},w_{1}^{n}\right)$ are jointly typical and $\left(\left(x_{1}^{n},y^{n}\right),x_{2}^{n},w_{2}^{n}\right)$ are jointly typical. Then, using the Markov chain $W_{1}-\left(X_{1},X_{2},Y\right)-W_{2}$, we can demonstrate that $\left(w_{1}^{n},w_{2}^{n},x_{1}^{n},x_{2}^{n},y^{n}\right)$ are joint typical. For a fixed $\left(x_{1}^{n},x_{2}^{n},x_{3}^{n},y^{n}\right)\in A_{\epsilon }^{*\left(n\right)}$, $W_{1}^{n}$ is drawn from $p(w_{1}|x_{1})$, which implies that $\left(x_{1}^{n},W_{1}^{n}\right)\in A_{\epsilon }^{*\left(n\right)}$ for sufficiently large *n*. $W_{2}^{n}$ is drawn from $p(w_{2}|x_{2})$, which implies that $\left(x_{2}^{n},W_{2}^{n}\right)\in A_{\epsilon }^{*\left(n\right)}$ for sufficiently large *n*; $W_{3}^{n}$ is drawn from $p(w_{3}|x_{3})$, which implies that $\left(x_{3}^{n},W_{3}^{n}\right)\in A_{\epsilon }^{*\left(n\right)}$ for sufficiently large *n*. Then, with high probability, $\left(W_{1}^{n},W_{2}^{n},W_{3}^{n},x_{1}^{n},x_{2}^{n},x_{3}^{n},y^{n}\right)\in A_{\epsilon }^{*\left(n\right)}$. □

**Proof of Theorem 1.** For m=1,2,3, fix $p\left(w_{m}|x_{m}\right)$ and $g(W_{1},W_{2},W_{3},Y)$ such that the distortion constraint $Ed(T,\hat{T})⩽D$ is satisfied. Calculate $p\left(w_{m}\right)=\sum_{x_{m}}p\left(x_{m}\right)p\left(w_{m}|x_{m}\right)$. □

Generation of codebooks: Generate $2^{nR_{m}^{\prime }}$ i.i.d codewords ${w_{m}}^{n}\left(s_{m}\right)∼\prod_{i=1}^{n}p\left(w_{m,i}\right)$, and index them by $s_{m}\in \left\{1,2,\ldots ,2^{nR_{m}^{\prime }}\right\}$. Provide $2^{nR_{m}}$ random bins with indices $t_{m}\in \left\{1,2,\ldots ,2^{nR_{m}}\right\}$. Randomly assign the codewords ${w_{m}}^{n}\left(s_{m}\right)$ to one of $2^{nR_{m}}$ bins using a uniform distribution. Let $B_{m}\left(t_{m}\right)$ denote the set of codeword indices $s_{m}$ assigned to bin index $t_{m}$.

Encoding: Given a source sequence $X_{m}^{n}$, the encoder looks for a codeword $W_{m}^{n}\left(s_{m}\right)$ such that $\left(X_{m}^{n},W_{m}^{n}\left(s_{m}\right)\right)\in A_{\epsilon }^{*\left(n\right)}$. The encoder sends the index of the bin $t_{m}$ in which $s_{m}$ belongs.

Decoding: The decoder looks for a pair $\left(W_{1}^{n}\left(s_{1}\right),W_{2}^{n}\left(s_{2}\right),W_{3}^{n}\left(s_{3}\right)\right)$ such that $s_{m}\in B_{m}\left(t_{m}\right)$ and $\left(W_{1}^{n}\left(s_{1}\right),W_{2}^{n}\left(s_{2}\right),W_{3}^{n}\left(s_{3}\right),Y^{n}\right)\in A_{\epsilon }^{*\left(n\right)}$. If the decoder finds a unique triple $\left(s_{1},s_{2},s_{3}\right)$, he then calculates ${\hat{T}}^{n}$, where ${\hat{T}}_{i}=g\left(W_{1,i},W_{2,i},W_{3,i},Y_{i}\right)$.

Analysis of the probability of error:

1. The encoders cannot find the codewords $W_{m}^{n}\left(s_{m}\right)$ such that $\left(X_{m}^{n},W_{m}^{n}\left(s_{m}\right)\right)\in A_{\epsilon }^{*\left(n\right)}$. The probability of this event is small if

(A3) $$\begin{array}{c} {R_{m}}^{\prime }>I\left(X_{m},W_{m}\right) \end{array}$$

2. The pair of sequences $\left(X_{1}^{n},W_{1}^{n}\left(s_{1}\right)\right)\in A_{\epsilon }^{*\left(n\right)}$, $\left(X_{2}^{n},W_{2}^{n}\left(s_{2}\right)\right)\in A_{\epsilon }^{*\left(n\right)}$ and $\left(X_{3}^{n},W_{3}^{n}\left(s_{3}\right)\right)$$\in A_{\epsilon }^{*\left(n\right)}$ but the codewords $\left\{W_{1}^{n}\left(s_{1}\right),W_{2}^{n}\left(s_{2}\right),W_{3}^{n}\left(s_{3}\right)\right\}$ are not jointly typical with the side information sequences $Y^{n}$, i.e., $\left(W_{1}^{n}\left(s_{1}\right),W_{2}^{n}\left(s_{2}\right),W_{3}^{n}\left(s_{3}\right),Y^{n}\right)\notin A_{\epsilon }^{*\left(n\right)}$. We have assumed that

(A4) $$\begin{array}{cc} \; & p\left(w_{1},w_{2},w_{3},x_{1},x_{2},x_{3},y\right)\\ & =p\left(w_{1}|x_{1}\right)p\left(w_{2}|x_{2}\right)p\left(w_{3}|x_{3}\right)p\left(x_{1},x_{2},x_{3},y\right). \end{array}$$

Hence, by the Markov lemma, the probability of this event goes to zero if *n* is large enough.

3. There exists another $s^{\prime }$ with the same bin index that is jointly typical with the side information sequences. The correct codeword indices are denoted by $s_{1}$ , $s_{2}$ and $s_{3}$. We first consider the situation where the codeword index $s_{1}$ is in error. The probability that a randomly chosen $W_{1}^{n}\left({s^{\prime }}_{1}\right)$ is jointly typical with $\left(W_{2}^{n}\left(s_{2}\right),W_{3}^{n}\left(s_{3}\right),Y^{n}\right)$ can be bounded as

(A5) $$\begin{array}{c} \begin{array}{cc} \; & \mathrm{Pr}\left\{\left(W_{1}^{n}\left(s_{1}^{\prime }\right),W_{2}^{n}\left(s_{2}\right),W_{3}^{n}\left(s_{3}\right),Y^{n}\right)\in A_{\epsilon }^{*\left(n\right)}\right\}\\ & ⩽2^{-n\left(I\left(W_{1};W_{2},W_{3},Y\right)-3\epsilon \right)}. \end{array} \end{array}$$

The probability of this error event is bounded by the number of codewords in the bin $t_{1}$ times the probability of joint typicality

(A6) Pr∃s1′∈B1t1,s1′≠s1:W1ns1′,W2ns2,W3ns3,Yn∈Aϵ*n⩽∑s1′≠s1,s1′∈B1t1PrW1ns1′,W2ns2,W3ns3,Yn∈Aϵ*n⩽2nR1−R1′2−nIW1;W2,W3,Y−3ϵ.

Similarly, the probability that the codeword index $s_{2}$ or $s_{3}$ is in error can be bounded by

(A7) $$\begin{array}{cc} \; & \mathrm{Pr}\left\{\exists s_{2}^{\prime }\in B_{2}\left(t_{2}\right),s_{2}^{\prime }\neq s_{2}:\right.\\ \; & \left.\left(W_{1}^{n}\left(s_{1}\right),W_{2}^{n}\left(s_{2}^{\prime }\right),W_{3}^{n}\left(s_{3}\right),Y^{n}\right)\in A_{\epsilon }^{*\left(n\right)}\right\}\\ & ⩽2^{n\left(R_{2}-R_{2}^{\prime }\right)}2^{-n\left(I\left(W_{2};W_{1},W_{3},Y\right)-3\epsilon \right)},\\ \; & \mathrm{Pr}\left\{\exists s_{3}^{\prime }\in B_{3}\left(t_{3}\right),s_{3}^{\prime }\neq s_{3}:\right.\\ \; & \left.\left(W_{1}^{n}\left(s_{1}\right),W_{2}^{n}\left(s_{2}\right),W_{3}^{n}\left(s_{3}^{\prime }\right),Y^{n}\right)\in A_{\epsilon }^{*\left(n\right)}\right\}\\ & ⩽2^{n\left(R_{3}-R_{3}^{\prime }\right)}2^{-n\left(I\left(W_{2};W_{1},W_{3},Y\right)-3\epsilon \right)}. \end{array}$$

We then consider the case that two of the three codeword indices are in error. The probability that the randomly chosen $W_{1}^{n}\left({s^{\prime }}_{1}\right)$ and $W_{2}^{n}\left({s^{\prime }}_{2}\right)$ are jointly typical with $\left(W_{3}^{n}\left(s_{3}\right),Y^{n}\right)$ can be bounded as

(A8) $$\begin{array}{cc} \; & \mathrm{Pr}\left\{\left(W_{1}^{n}\left(s_{1}^{\prime }\right),W_{2}^{n}\left(s_{2}^{\prime }\right),W_{2}^{n}\left(s_{3}\right),Y^{n}\right)\in A_{\epsilon }^{*\left(n\right)}\right\}\\ & =\sum_{\left(W_{1},W_{2},W_{3},Y\right)\in A_{\epsilon }^{*\left(n\right)}}p\left(w_{1}^{n}\right)p\left(w_{2}^{n}\right)p\left(w_{3}^{n},y^{n}\right)\\ & ⩽2^{-n\left(I\left(W_{1};W_{2},W_{3},Y\right)+I\left(W_{2};W_{1},W_{3},Y\right)-I\left(W_{1};W_{2}|W_{3},Y\right)-4\epsilon \right)}. \end{array}$$

Hence, the error probability can be bounded as

(A9) $$\begin{array}{cc} \; & \mathrm{Pr}\left\{\exists s_{1}^{\prime }\in B_{1}\left(t_{1}\right),s_{1}^{\prime }\neq s_{1},\exists s_{2}^{\prime }\in B_{2}\left(t_{2}\right),s_{2}^{\prime }\neq s_{2}:\right.\\ \; & \left.\left(W_{1}^{n}\left(s_{1}^{\prime }\right),W_{2}^{n}\left(s_{2}^{\prime }\right),W_{2}^{n}\left(s_{3}\right),y^{n}\right)\in A_{\epsilon }^{*\left(n\right)}\right\}\\ \; & ⩽2^{n(R_{1}-R_{1}^{\prime }+R_{2}-R_{2}^{\prime })}\\ \; & \times 2^{-n\left(I\left(W_{1};W_{2},W_{3},Y\right)+I\left(W_{2};W_{1},W_{3},Y\right)-I\left(W_{1};W_{2}|W_{3},Y\right)-4\epsilon \right)}. \end{array}$$

Similarly, we can obtain the probability that the codeword indices $\left(s_{1},s_{3}\right)$ or $\left(s_{2},s_{3}\right)$ are in error, which we omit here.

For the case where all the codeword indices $s_{1}$, $s_{2}$ and $s_{3}$ are in error, the probability that the randomly chosen $W_{1}^{n}\left({s^{\prime }}_{1}\right)$, $W_{2}^{n}\left({s^{\prime }}_{2}\right)$ and $W_{3}^{n}\left({s^{\prime }}_{3}\right)$ are jointly typical with $Y^{n}$ can be bounded as

(A10) $$\begin{array}{c} \begin{array}{cc} \; & \mathrm{Pr}\left\{\left(W_{1}^{n}\left(s_{1}^{\prime }\right),W_{2}^{n}\left(s_{2}^{\prime }\right),W_{2}^{n}\left(s_{3}^{\prime }\right),Y^{n}\right)\in A_{\epsilon }^{*\left(n\right)}\right\}\\ & =\sum_{\left(W_{1},W_{2},W_{3},Y\right)\in A_{\epsilon }^{*\left(n\right)}}p\left(w_{1}^{n}\right)p\left(w_{2}^{n}\right)p\left(w_{3}^{n}\right)p\left(Y^{n}\right)\\ & ⩽2^{-n\left(I\left(W_{1};W_{2},W_{3},Y\right)+I\left(W_{2};W_{1},W_{3},Y\right)\right)}\\ \; & \times 2^{-n\left(I\left(W_{3};W_{1},W_{2},Y\right)-I\left(W_{1};W_{2}|W_{3},Y\right)-I\left(W_{1},W_{2};W_{3}|Y\right)-5\epsilon \right)}. \end{array} \end{array}$$

The probability of the above error events goes to 0 when

$$\begin{array}{c} \begin{array}{cc} R_{1}^{\prime }-R_{1} & ⩽I\left(W_{1};W_{2},W_{3},Y\right)\\ & \ldots \end{array} \end{array}$$

(A11) $$\begin{array}{c} \begin{array}{cc} R_{1}^{\prime }-R_{1}+R_{2}^{\prime }-R_{2}+R_{3}^{\prime }-R_{3} & ⩽I\left(W_{1};W_{2},W_{3},Y\right)\\ & +I\left(W_{2};W_{1},W_{3},Y\right)\\ & +I\left(W_{3};W_{1},W_{2},Y\right)\\ & -I\left(W_{1};W_{2}|W_{3},Y\right)\\ & -I\left(W_{1},W_{2};W_{3}|Y\right). \end{array} \end{array}$$

Therefore, (2) can be obtained by combining (A3) and (A11).

If $\left(s_{1},s_{2},s_{3}\right)$ are correctly decoded, we have $\left(X_{1}^{n},X_{2}^{n},X_{3}^{n},W_{1}^{n}\left(s_{1}\right),W_{2}^{n}\left(s_{2}\right),W_{3}^{n}\left(s_{3}\right),Y^{n}\right)\in A_{\epsilon }^{*\left(n\right)}$. Therefore, the empirical joint distribution is close to the distribution $p\left(w_{1}|x_{1}\right)$$p\left(w_{2}|x_{2}\right)$$p\left(w_{3}|x_{3}\right)p\left(x_{1},x_{2},x_{3},y\right)$ that achieves distortion *D*.

## Appendix B

Here, we provide the rigorous proof of Theorem 2. Define a series of encoders $f_{m}:X_{m}^{n}\to \left\{1,\ldots ,2^{nR_{m}}\right\}$ and decoders $g:\prod_{m\in M}\left\{1,\ldots ,2^{nR_{m}}\right\}\times Y^{n}\to {\hat{T}}^{n}$ that achieve the given distortion *D*. We can derive the following inequalities

nR1≥H(f1(X1n))(A12a)≥H(f1(X1n)|f2(X2n),f3(X3n),Yn)≥H(f1(X1n)|f2(X2n),f3(X3n),Yn)−H(f1(X1n)|f2(X2n),f3(X3n),Yn,X1n,X2n,X3n)(A12b)=I(X1n,X2n,X3n;f1(X1n)|f2(X2n),f3(X3n),Yn)=∑i=1nI(X1,i,X2,i,X3,i;f1(X1n)|f2(X2n),f3(X3n),(A12c)Yn,X1,1i−1,X2,1i−1,X3,1i−1)=∑i=1nH(X1,i,X2,i|f2(X2n),Yn,X1,1i−1,X2,1i−1)(A12d)−H(X1,i,X2,i|f1(X1n),f2(X2n),Yn,X1,1i−1,X2,1i−1)(A12e)=∑i=1nH(X1,i,X2,i|W2,i,Yi)−H(X1,i,X2,i|W1,i,W2,i,Yi)=∑i=1nI(X1,i,X2,i;W1,i|W2,i,Yi),

where (A12a) follows from the fact that conditioning reduces entropy, (A12b) and (A12d) are obtained by the definition of conditional mutual information and (A12c) is the chain rule of mutual information. In (A12e), we let $W_{1,i}=\left(f_{1}\left(X_{1}^{n}\right),X_{1,1}^{i-1},X_{2,1}^{i-1},Y_{1}^{i-1},Y_{i+1}^{n}\right)$, and $W_{2,i}=\left(f_{2}\left(X_{2}^{n}\right),X_{1,1}^{i-1},X_{2,1}^{i-1},Y_{1}^{i-1},Y_{i+1}^{n}\right)$. Similarly, we have

(A13) $$\begin{array}{c} nR_{2}\geq \sum_{i=1}^{n}I\left(X_{1,i},X_{2,i},X_{3,i};W_{2,i}|W_{1,i},W_{3,i},Y_{i}\right),\\ nR_{3}\geq \sum_{i=1}^{n}I\left(X_{1,i},X_{2,i},X_{3,i};W_{3,i}|W_{2,i},W_{3,i},Y_{i}\right). \end{array}$$

For the sum rate part, we have

n(R1+R2)≥H(f1(X1n),f2(X2n))(A14a)≥H(f1(X1n),f2(X2n)|Yn)≥H(f1(X1n),f2(X2n)|Yn)−H(f1(X1n),f2(X2n)|Yn,X1n,X2n)(A14b)=I(f1(X1n),f2(X2n);X1n,X2n|Yn)(A14c)=∑i=1nI(f1(X1,i),f2(X1,i);X1n,X2n|Yn,X1i−1,X2i−1)=∑i=1nH(X1,i,X2,i|Yn,X1i−1,X2i−1)(A14d)−H(X1,i,X2,i|f1(X1n),f2(X2n),X1,1i−1,X2,1i−1,Yn)=∑i=1nH(X1,i,X2,i|Yi)(A14e)−H(X1,i,X2,i|f1(X1n),f2(X2n),X1,1i−1,X2,1i−1,Yn)(A14f)=∑i=1nH(X1,i,X2,i|Yi)−H(X1,i,X2,i|W1,i,W2,i,Yi)=∑i=1nI(X1,i,X2,i;W1,i,W2,i|Yi),

where (A14a) follows from the fact that conditioning reduces entropy, (A14b) and (A14d) are obtained by the definition of conditional mutual information, (A14c) is the chain rule of mutual information and (A14e) follows from the fact that $X_{1,i},X_{2,i}$ is conditionally independent of the past and feature of $X_{1},X_{2},Y$ given $Y_{i}$. In (A14f), the definition of $W_{1,i},W_{2,i}$ is used again.
